# Surgery

**Published:** 1825-10-01

**Authors:** 


### Surgery.

1. *Intus-susception, Operation for.* In Hufeland's Journal, for February of the present year, there is a very interesting case narrated; and, if strictly authentic, it becomes an important document. The patient was a strong man, who, while collecting wood in the district of Olpe, felt, all at once, a dragging pain in the region of the umbilicus, which quickly augmented, and forced him to cease working. He crawled home with difficulty, and laid himself down to rest. On awaking from repose, he vomited some slime, and the pain had decreased. In the evening, he had a motion. Next day, the patient was worse, being afflicted with colicky pains, recurring every 20 or 30 minutes, with intervals of ease. *Castor oil, salts, venesection, prescribed by the parish doctor.* Temporary relief only was obtained, and the pains returned in the evening, with increased violence, and much discharge of wind. The symptoms continued much the same for the next four days, when Dr. Fuschstius was consulted. He found the patient in bed, and not then in pain. His countenance was pale, and tinged slightly yellow, with an expression of anxiety in his looks. The temperature of the body was not much increased—the abdomen was not distended nor hot to the feel—but it was sensible on pressure—pulse regular, soft, and about 60 in the minute—an induration about the angle formed by the ascending and transverse portions of the colon. This hardened spot was unequal on its surface, feeling like an uneven, distended intestine. The patient had had no stool for five days, and had vomited only once. In half an hour after Dr. F's arrival, the colick pains came on, and the patient was obliged to run up and down the room in torment. During this time, something hard, about the size of a fist, could be felt in the place abovementioned, and this spot was the chief seat of pain. The idea struck our author, that the complaint arose from mechanical obstruction. He directed the venesection to be repeated, and leeches to be applied to the part. A strict antiphlogistic plan was pursued, till the eighth day of the disease, while baths, fomentations, glysters, &c. were tried, without any good effect. The patient's strength was considerably reduced, and his spirits depressed. The part remained indurated, as before. Dr. F. become more and more convinced that there was either a volvulus, or an intus-susception of the gut, and he stated to the friends, that, without an operation, there was no hope of recovery. As the operation was not immediately consented to, Dr. F. changed his mode of treatment. He gave two or three spoonfuls of warm red oil, and a grain of opium every hour, for six hours consecutively. This treatment produced longer intervals from pain. Large quantities of cold water were thrown up the rectum, in a continued stream, but they quickly returned. The patient felt that the water reached the hardened part, but passed no farther. No abatement of the disease took place. On the ninth day, vomiting occurred, of matters having a bad, but not stercoraceous smell. The abdomen was now greatly distended, and the indurated part remained in the same state and place. Mercury was given in the quantity of six ounces at a time. Vomiting immediately followed the exhibition of the medicine, but no quicksilver was ejected. No passage downwards, however, was effected. The operation was, at last, consented to, and performed on the 10th day of the disease.

*Operation.* It was determined to open the abdomen, at the outer edge of the right rectus muscle, two inches above the level of the umbilicus. The opening was effected in the usual way, and to the extent of about two or three inches. The operator then smeared his hand with oil, and introduced it into the abdomen, in order to search for the indurated part. An attack of colick now supervened, and forced a portion of intestine through the wound, but it was immediately replaced by an assistant. On farther examination, Dr. F. discovered in the ileum a foreign substance just where the hardened part had been felt externally. He drew the intestine out, in order to examine it more minutely. The intestine was neither inflamed nor distended, but contained in its cavity a soft compact mass, which could be felt as far as he could follow the intestine. He distinctly ascertained an intus-susception, but could not reach the commencement of it, so as to bring it out. Our author determined to open the intestine itself, rather than enlarge the opening in the abdominal parietes. The gut was, therefore, incised at one end of the intus-susception, and immediately a portion of the invaginated intestine came in view. The operator introduced his finger into the opening, which was about two inches in length, and gradually pushed back the intus-suscepted part from the right to the left side, while he gently drew that part of the intestine containing the intus-susception towards him. By this procedure, he fortunately succeeded in unfolding the entangled intestine, *which amounted to two feet in length*! There was no trace of inflammation, or any of its consequences to be seen. There was a teres worm in the upper part of the invagination. No remains of the quicksilver could be found. The wound in the intestine was brought together by means of six stitches, in the form of the glover's suture, the ends of the silk thread being brought out of the wound. The integuments were next secured by the interrupted suture. The patient was then put to bed, and some broth given him. Nothing was administered to the patient for some days, but broth and gruel alternately. No colick pains occurred after the operation, and a natural stool took place on the second day. By the 14th day, the patient was completely cured, and remains well.

The foregoing operation has made a strong impression on our minds, in consequence of a melancholy and interesting case which we lately attended, in company with Dr. Richardson, Dr. Outran, Sir A. Carlisle, and Mr. Davies. The patient was a young man, who had travelled with the late Mr. Belzoni, and was possessed of extraordinary capacity for the acquisition of languages, and the facility with which he personated the characters of people of different nations. He had had a severe abdominal inflammation while in Egypt, some years ago, and since that period had been very irregular in his bowels, and subject to long-continued constipation. In the month of May last he was seized, after lifting a heavy weight, (a piece of an Egyptian statue, in Leicester Square) with pain in the abdomen, attended with obstinate constipation, which could never be overcome till death, which took place ten or twelve days from the commencement of the disease. Every means were tried, during that period, including the exhibition of quicksilver, but without the slightest good effect. Vomiting of stercoraceous matters—great distention of the abdomen—dreadful colicky pains—and all the phenomena of ileus occurred, and continued many days before death, producing altogether one of the most distressing scenes which we ever witnessed!

The body was examined by Mr. Alcock, in the presence of several medical gentlemen. The intestines, above the obstruction, were greatly distended; but there was no inflammation that could fairly account for the patient's death. A portion of ileum had got entangled under an old band or *bride*, formed probably during the illness in Egypt, and was easily drawn out; but a complete obstruction—in fact, a regular internal strangulated hernia had been the consequence. One or two trifling intus-susceptions were seen in the neighbourhood. We have not the smallest doubt, that an operation would have readily freed the intestine from this strangulation, and that the only danger would have been simply that of gastrotomy. There would have been no necessity for opening into the cavity of the intestine itself.

In respect to the German operation above described, we think it will be admitted by our surgical readers, that it would be much safer to enlarge the opening in the abdominal parietes, than, from want of room, be compelled to slit open the intestine itself. This last part of the operation is surely a fearful addition to the quantum of danger run in such a venturesome proceeding.

The records of medicine present little, if any thing, that can enlighten us on the subject of the operation in question. Our author informs us, that Nuck performed a similar operation, as recorded in Haller's Disputations. We have searched for the case, but could not find it. It is the only successful instance which he could find in the annals of medicine. If a German say so, we will search no farther. The late operations of Mr. Lizars, together with the case of Dr. F. himself, and the post mortem appearances in the case of Mrs. Belzoni's servant, will probably give more confidence to the future operator, than any thing which can be raked up from the records of antiquity.

---

2. *Lithotomy.* Mr. Charles Averill has lately performed the operation in the manner recommended by Mr. Aston Key, as described in our first number (new series) p. 206, *et seq.* A straight staff was introduced into the bladder, and an incision made in the usual manner, till the groove was exposed. The same knife was carried along the groove, in the manner directed by Mr. K. and the prostate gland divided throughout its whole extent. Then directing his nail along the groove in the staff, the operator felt his way into the bladder, till the point of his finger came against the rough surface of the stone, when he withdrew the staff, and, having used his finger as a conductor for the forceps, he withdrew it also. He seized the stone, but could not extract it, as the wound was too small. This was enlarged, by means of Sir Astley Cooper's beaked knife. The forceps being again introduced, the stone was extracted. It was of the mulberry species, and weighed one ounce and a quarter, the patient being a boy of nine years of age.

---

3. *Phlegmatia Dolens.* Mr. Davies has stated a case of this affection terminating in sphacelus, and requiring amputation of the limb. Mrs. B. the mother of ten children, had her lower extremities much swelled previously to her accouchement. A tremendous hæmorrhage succeeded delivery, but ceased with the expulsion of the placenta. In four or five days, Mr. Davies took his leave. This was on the 28th of April. On the 5th of May, she again sent for Mr. Davies, and complained of much weakness and relaxation of bowels. These symptoms being removed by appropriate remedies, the patient was suddenly seized, on the 8th of May, with a most excruciating pain in the left loin and hip, which suddenly flew down into the leg and foot. The limb became instantly paralysed, from below the knee to the toes. When Mr. D. arrived, the leg and foot had lost all sensation and power of motion; it also felt cold. "The limb was not evidently swelled, but there was a good deal of tenderness in and about the ham." The pulse was from 150 to 160, and extremely feeble—the countenance was expressive of pain and misery. Laudanum was exhibited, in pretty large doses, and gave considerable relief. On the morning of the 9th, (placed the 6th in the statement) the member was a good deal swelled, and the leg and foot completely cold and insensible. On the 10th, the foot and lower part of the leg were purple. On the 11th, much the same as before. Mr. Bransby Cooper saw the patient. There were no positive proofs of sphacelus. The sulphate of quinine was ordered every four hours. Late in the evening, however, some vesicles appeared on the back of the foot, and indicated mortification at hand.

Things went on for several days, and at last a considerable sloughing took place on the lower and back part of the leg, so as to expose the tendo achillis. In consultation, it was deemed advisable to amputate. As soon as the vessels were cut across, the blood in the veins appeared coagulated. Much serum oozed from the stump, when the leg was removed. The thigh of the affected side was, at this time, nearly double the size of the other.

When the arteries of the amputated limb were injected, the wax ran freely out at the sloughing ends of the vessels. The arteries appeared healthy above the line of demarcation. The veins were completely distended with firmly coagulated blood. Their coats were thickened, and their inner surfaces very much inflamed. The small veins were in a state similar to that of the large ones. The nerves were sound. "The morbid appearances tend to confirm the truth of Dr. Davis's views of the pathology of phlegmasia dolens." As the patient, however, has recovered, there is no means of ascertaining, at present, how far the inflammation and thickening of the veins proceeded upwards, or whether the coagulation of their contents was owing to a mechanical or vital cause.

For our own parts, we do not consider the disease as one of well-marked phlegmasia dolens at all—at least, it does not correspond with the cases which we have seen, about three or four in number. The symptoms, and the termination were very different from those usually observed in the disease under whose name it is ranked by Mr. Davies. We have no fault, however, to find with that gentleman's mode of treatment or management of a formidable affection of very anomalous character.—*Repos. June,* 1825.

P. S. Two or three days after the date of last report, Mr. D. informs us, in a subsequent No. of the Repository, that the patient was seized with a violent shivering, and nearly died in the attack. By a great quantity of stimulants, she was recruited ; but another fit came on, the succeeding day, and terminated her existence. On dissection, "the femoral vein of the affected side was inflamed in the highest degree, and its coats thickened. It was full of coagulated blood. This state of the vein extended throughout the iliac into the cava, nearly as high as the diaphragm. The disease of the vein did not descend at all on the opposite side from its bifurcation. All the small veins, as far as we could perceive, of the diseased limb, were in a similar state ; but we must infer that some of them were pervious, otherwise the blood could not have returned from the arteries. The inner coats of the arteries were red throughout the body."

In the same No. of the Repository, Dr. Davis relates a few particulars of a case of phlegmasia dolens, in the person of a medical gentleman. He was attacked, on the 10th of February, 1825, whilst in the last stage of phthisis, in the right thigh and leg, the affection being perfectly characteristic, says Dr. Davis, of phlegmasia dolens. "The whole limb was uniformly enlarged and extremely painful up to Poupart's ligament. It had a dull white appearance, and exhibited no indentation from pressure." On dissection, "the right common internal and external iliac veins seemed unusually prominent, and appeared to the touch, of a firm consistence like cord. Upon opening these veins, they were found to be filled with layers of coagulable lymph, so as to render them completely impervious. This state of disease was traced as far as the upper part of the femoral vein." The corresponding arteries were healthy.

This appears to have been a very different affection from that described by Mr. Davies.

---

4. *Paracentesis Thoracis.** Dr. Jackson, of Philadelphia, has lately published a case where paracentesis was performed, and where we observe several interesting pathological features. The patient was a Negro servant, who presented himself to Dr. J. labouring under inflammation of the right lung, the left being unaffected. By proper means the severity of the attack was soon subdued, but some cough still continued, and the patient being anxious to attend to his domestic duties neglected his complaint. About two months afterwards, (middle of October, 1824) he had another attack of acute pneumonia in the same side, for which he was again actively treated. On the 30th of October, he was seized with pleuritis of the *left* side. "Examination, by percussion, showed the lower portion of the thorax, on the left side, to want resonance: it returned a very flat, dull sound, like solid flesh. Above the fourth rib, it was unnaturally resonant—it sounded as though perfectly hollow. With the stethoscope, the total absence of respiration in the left lung was determined, not only in front, but under the axilla, and between the scapula and spine, except very high up, where it was indistinct. I could not discover hægophonism, but metallic tinkling, or a sound resembling that produced by striking a deep silver or glass vase, was very perceptible. These signs were satisfactory evidence that an effusion, both of liquid and air, had already taken place into the sac of the left pleura. From the absence of any loud sibilant rattle, I was satisfied the air was not derived from a communication with the lungs, and that the effusion did not consist of pus, proceeding from an abscess that had ruptured in the lungs, but was serum, the result of acute inflammation of the pleura." 121. It is not necessary here, to detail the treatment which was pursued, being that properly employed in pneumonia and pleurisy. By the 10th November, all the acute symptoms had subsided, and a free and copious expectoration of thick viscid mucus was established. The respiration was free—the skin soft and moist. An exploration of the left side presented no other alteration than a disappearance of the metallic tinkling. In the right lung, a mucous and slight crepitous rattle still existed. Towards the end of November, œdema of the lower extremities was observed, with hurried respiration, and breathlessness on ascending stairs. Exploration by the stethoscope discovered the following phenomena.

"At rest, the respiration was natural, but laborious and oppressive on motion—cough not very troublesome, loose, and expectoration free—decubitus perfectly easy on either side—œdema of left hand and foot—above the fourth rib, on the left side, the chest yielded, on percussion, a resonance unnaturally clear and loud—below the fourth rib, unnaturally flat and dead—on the right side, the resonance

---

* Philadelphia Journal, No. 1, New Series.

was natural, though chiller in the upper part than in the corresponding portion of the left, and clearer in the lower than in the same region of the opposite side. Examined with the stethoscope, the murmur of respiration was heard in every part of the right lung, louder than natural, and with a faint crepitous rattle—in the left lung, no respiration could be discerned in any part. From these evidences, no doubt would be entertained, but that pneumo-thorax, or an effusion of air, and a serous effusion existed in the left side, the effects of inflammation of the pleura, which had become chronic, together with irritation, or chronic inflammation of the right lung. To give greater certainty to the diagnosis, the chest was measured, and the left side ascertained to be an inch and a half larger than the right side. Succussion was also performed, when the splash of a liquid in the chest, was to be distinctly heard, even at the distance of a foot or two from the patient. The sound and motion were both very evident to the patient himself. This last phenomenon, when it occurs, is the most unequivocal mark of effusion in the thorax, but it is not present in all cases. It is only to be met with when an effusion of air, as well as serous fluid, has conjointly taken place." 122.

It is abundantly evident that, under such circumstances, an operation presented the only chance of recovery—and even *that* offered but a faint one. It was performed on the 15th of December, by Dr. Gibson, in the presence of a class of pupils, at the Alms-house Infirmary, and several other medical gentlemen. The incision was made between the sixth and seventh ribs, at the point of their greatest projection. Two quarts of clear serous fluid, containing some flocculi of coagulated fibrin, were drawn off. The only inconvenience which appeared to result during the operation, was a severe fit of coughing, from the introduction of the gum-elastic catheter. But when the fluid was drawn off, pain was complained of, and eight ounces of blood were abstracted. In the evening, there being some reaction, twelve ounces of blood were taken from the arm, and a rigid diet prescribed. Next morning, the symptoms were relieved—the respiration was no longer embarrassed—no sense of suffocation—pain nearly gone. Diuretics and cupping were prescribed, and, under this treatment, the symptoms gradually subsided, excepting the cough, which still continued troublesome, and the sputa evinced a suspicious character.

"On the 5th of January, the following was ascertained to be the state of the chest. Left side, resonant throughout its whole extent; the incision had united by first intention, but had not cicatrized; no respiratory murmur was to be distinguished, in any part. Right side, natural resonance on percussion, except the lower portion, where it was, in a slight degree, flatter than natural; respiration puerile, or louder than is usual in adults, crepitous rattle, and, occasionally, mucous rattle in superior and middle lobe; respiration fainter in lower lobe. The left side was a quarter of an inch smaller than the right, and the left shoulder two inches lower than the opposite.

"From these circumstances, the following conclusions were drawn. 1st. No serous effusion had again occurred on the left side. 2d. The left lung had not expanded, but remained compressed, by the air contained in the pleura, or from a change in structure. 3d. Irritation, and sub-inflammation of mucous membrane of bronchiæ, and of the parenchyma of right lung existed, which, from its obstinacy, it might be apprehended, was maintained by the development of tubercles, and consequently, that symptoms of phthisis pulmonalis would soon make their appearance. 4th. That the lower lobe of the right lung was more congested than the upper, or that a slight effusion existed in right pleura. Ungt. antimon. tart. was directed to both sides of the chest, and strict regimen." 125.

By the 16th of January, the pain had become permanent, and the respiration embarrassed. The *left* side was now found to return a dull dead sound, on percussion, below the fourth rib—a clear and hollow sound above. The right side was less resonant than natural in its lower portion, where acute pain was also felt. On succussion, the splash of a fluid was again audible. From this time till the 10th of February, the symptoms fluctuated much. On the latter date, during a violent paroxysm of coughing, the wound in the left side, which had not completely cicatrized, burst open, with an explosion, and gave vent to a considerable quantity of clear serum, containing coagulated flocculi. The discharge gave relief to the feelings of oppression. The right side of the chest gradually lost its resonance, "indicating the progress of tuberculous degeneration"—œdema of the face and extremities supervened—hectic fever commenced—pectoriloquism was detected in the upper lobe of the right lung—hæmoptysis ensued, and death took place on the 19th March.

"The morning of 20th, the body was examined by Dr. Gwinner, the resident house physician, and myself, when the following were the appearances observed. Emaciation almost complete; infiltration universal. On raising the sternum, the right lung did not collapse; the left side of the thorax presented a cavity. The right pleura was reddened with vessels, where lining the diaphragm, a few adhesions existed, and about a gill of effused serum. The lung felt solid. When laid open, numerous tubercles were displayed, some immature, others softened, and some were evacuated, leaving cavities. In the upper lobe, where pectoriloquism had been detected, was a cavity, which could have contained a walnut. In the centre of the lower lobe was another cavity still larger. The whole lung was infiltrated with serous mucus, and purulent fluid.

"The left lung was compressed against the mediastinum, and not more than a fourth of its natural volume. It contained a few tubercles, none in a state of suppuration, and no cavity. It was rendered solid by the long compression it had undergone, but not disorganised nor changed in structure. The pleura pulmonalis was covered with a thick coating of coagulated fibrin. The pleura had generally a similar coat, but not as thick; that covering the diaphragm, was of a deeper red, and tuberculated." 126.

It is evident that the condition of the lungs rendered an operation hopeless in this case; but it is highly probable, that had there been no tubercular tendency, the evacuation of the fluid might have afforded a considerable chance of recovery. The case, therefore, is not calculated to deter from a similar operation in future, and especially under more favourable circumstances.—*Editor*.

---

5. *Extirpation of the Tonsils.* Mr. Pettigrew has lately cut out both tonsils from the throat of a young girl twelve years of age. They had been enlarging from youth—and now were in contact, from their size, threatening suffocation. She had a strong nasal twang in her speech—was unable to swallow solid substances—and could only take fluid nutriment in small quantities. Iodine, internally and externally, was tried, but in vain. The knife was preferred to the ligature, and was thus used.

The patient was seated on a chair before the operator, her head firmly held by an assistant, and her tongue pressed down by a spoon. Mr. P. then planted a hook into the left tonsil, and drew it gently forward—then carrying a very fine probe-pointed bistoury to the back of the gland, he immediately cut it out. A violent hæmorrhage succeeded, but soon subsided. The other tonsil was then treated in the same manner. The surfaces whence the glands were removed, healed in a few days, and the little patient was relieved from the great inconvenience which she had so long suffered. The bistoury, Mr. Pettigrew thinks, is a much more safe and convenient instrument than the knife—and liable to no objection. "The operation I have recommended," says he, "is so exceedingly simple—so easy of performance—and so free from any dangerous result that I flatter myself it will be considered as an improvement in surgery." We would only observe that this operation is not new. Cooper traces the hook and bistoury to the time of Galen, as the then instruments for removing enlarged tonsils. The scalpel is that which is now used, but probably Galen's plan revived by Mr. Pettigrew, will be found the best. —*Med. Repos. May*, 1825.

---

6. *Treatment of Incised Wounds.** English surgeons pride themselves over those of France, in respect to the means of procuring adhesion by the first intention. Yet Mr. Symes apprehends that "our practice still continues to be tinctured with much superstition and barbarism." Every incised wound, he remarks, after being dressed according to the surgeon's fancy, is bound up with a long bandage tightly and curiously turned. And, although the patient soon complains of heat, pain, and oppression, all his intercessions for relief are of no avail. The bandage must not be disturbed till the fourth day, when the long-

* Mr. James Symes. Ed. Journal, July, 1825.

wished for, and much-dreaded dressing is to take place. Our author avers, that the object sought (adhesion by the first intention) is rarely obtained. When plasters and bandages are removed, "a gush of fetid pus for the most part" puts an end to the anticipations of union by the first intention. "Indeed," says Mr. S. "the French practice is much more prudent than our own: for, laying their account with the formation of pus, they provide a drain for its discharge, and thus prevent the formation of those artificial, and frequently extensive abscesses which so often result from injudicious attempts to obtain adhesion by the first intention." Our author seems confident that, in this description, he has not made matters worse than they really are. If *he* has not—we think the *students* have, in the following passage. "Many students have assured me that, in the whole course of their hospital attendance, they never saw an instance of union by the first intention—except in cases of cancer, harelip, or venesection." If this be the case in the hospitals of the North, the air wants purifying—or the art itself is defective. Numerous instances of union by the first intention may be seen in the hospitals of this country.

Adhesive plaster, he thinks, is the great enemy to adhesion, and as this was not invented in the time of Taliacotius, union by the first intention was more frequent then than now. The following are the plans recommended by Mr. Symes, and they are submitted to the judgment of the surgical reader.

"It is not necessary for the prevention of adhesion, that *blood* should be effused, since the uniting action is always, for the first day and part at least of the second, attended with copious discharge of thin serous matter, which acts quite as effectually in preventing the surfaces from adhering, that is to say, if they be of any considerable extent; for in very small wounds primary union happens, do what we will to oppose it.

"This copious and continued discharge of serous fluid, during the adhesive process, is what I am most anxious to impress upon the reader; for if it be kept in mind, there can be little difficulty in perceiving why the sealing up of wounds should be the most certain means of keeping them open, or in determining as to the best means for conducing to a different result.

"It is plain, that in the first instance no effort should be made to close the orifice completely—that pressure should be directed to the bottom rather than the outlet of the wound—that blood should not be allowed to crust over the lips—for though blood may be the best, that is the least hurtful balsam, I have often known it confine the serous discharges, and so prevent union—and that the orifice should be wiped and kept dry as long as it exudes any moisture.

"For fulfilling these indications, I would recommend, in the first place, that when means are required for keeping the cut edges in contact, stitches should be preferred to adhesive plaster, since their effect is exerted at considerable depth, while they offer little or no resistance to the exit of fluid. Some surgeons employ stitches and plasters together, but this plan I dislike; for plasters, acting only on the surface, always tend powerfully to close the lips, or even turn them inwards, while they not only effect no deep pressure, but even render the parts concerned more loose and disposed for the formation of sinuses; and stitches, if put in close enough, may always be rendered equivalent to the requisite contraction. It may be noticed, however, that a little gaping of the edges is by no means hurtful in the first instance, since it ensures a thorough discharge, and is rectified as soon as adhesion begins.

"Secondly, I would advise the disuse of all long, complicated bandages, which cannot be tightened or slackened in part, and removed without disturbing the patient.

"Compresses having been laid along the sides of the wound, it will be easy to effect pressure by very simple means, independently of long circular rollers. For instance, in wounds of the trunk, what can be more powerful or manageable than a broad piece of linen, but long enough to cross a little over the breast, and having its two ends torn longitudinally into three or four portions, and one of which may be tightened or slackened as occasion requires, while the whole may be thrown back at a moment's notice?

"For wounds of the extremities, a similar bandage, on a smaller scale, will answer equally well; and as for stumps, I beg to recommend a very simple contrivance, that has often saved me much trouble. A roller having been put on as usual, from above downwards, until it has come within a few inches of the stump, it should be fastened away; and then a few narrow strips of cloth being pinned or otherwise attached on each side, nothing remains to be done but tying them to each other across the face of the stump.

"Lastly, I would advise that the surgeon should apply dry caddis over the wound, as often as the least moisture is perceived.

"If these directions be observed, I feel satisfied, from my own experience, that union by the first intention will become an ordinary occurrence. And of how much consequence this is in all cases of wounds, but particularly in such as are complicated with fracture of bones, surgeons need not be told by me."

We believe there is some truth (as well as some caricature) in Mr. Symes's criticisms on the general mode of managing incised wounds—and that some benefit may be derived from the plan which he recommends in the foregoing extract.

---

7. *Perforations of the Soft Palate.** It appears that, till very lately, perforations of the velum pendulum palati, have seldom been remedied by artificial means. Yet they are very distressing accidents, not only as affecting the voice, but the power of deglutition. We, this day, (7th July) saw a fine young woman, whose *vel. pend. pal.* has been perforated by an ulcer, leaving an oblong opening which permits the egress of

* Mr. Allan. Ed Journal, July, 1825.

fluid and soft substances through the nose, when she attempts to swallow. The case narrated by Mr. Allan we have seen—and the simple remedy contrived by him appears to answer the purpose, at least for the present. The patient is a widow, 31 years of age, who applied to our author on the 1st July, 1824, to relieve her from a substance that was sticking in the œsophagus, which she described as a thin plate of silver, with a piece of sponge attached to it, and which had been adapted a few days previously with the view of closing an opening in the *vel. pend. palat.*, the consequence of an ulcer. The form of the aperture approached that of a triangle, the base being a quarter of an inch in length, and parallel to the margin of the velum. At this time the edges of the aperture were swelled and excoriated from the instrument applied.

Mr. A. first attempted to close the aperture by suture, having pared the edges for that purpose. A partial success was the consequence. Another suture was therefore passed, but it cut its way out, and did no good. A third attempt was made, and with equal want of success. The aperture now remained nearly of a circular form. Mr. Weiss next furnished our author with a stopper composed of two flat circular slices of India rubber, connected together by a neck of the same material, attached at either end to the centres of the circular pieces. The instrument then resembled an old-fashioned stud sleeve-button. The several parts, however, soon separated from the action of the saliva in the mouth. Our author then joined three pieces of the same material by means of a silk thread. The central piece sufficient to fill the aperture and the two other pieces overlapping the central one about the eighth of an inch all round, so that "when once one end was pinched up and introduced through the opening, it expanded behind the velum, and there was no probability of the instrument being displaced by any motion of the part." But the threads gave way, and then Mr. Allan shaped a new instrument, of the form last described, out of solid India rubber, making the overlapping parts convex towards each other, or where they are in contact with the velum, by which means irritation by friction was obviated. This apparatus had been in three weeks, at the date of report, and answered the purpose effectually.

Mr. Allan suggests a similar contrivance for defects in the bony palate. Mr. Weiss, it appears, has furnished a person labouring under such defect with one of these, and reports that it answers the purpose well.

There is one objection, however, to the obturator for the soft palate, and it is an important one, namely, that the perforation is likely to get larger by its use—a fact which we have reason to know. Again, we believe we may confidently state that these perforations have a constant tendency to diminish, when not prevented, and that the greater number of them, in time, become almost obliterated. This circumstance would induce us to recommend patience under the inconvenience, till the natural cure has a chance of being effected.—*Rev.*

8. *Sympathetic irritation of Urethra.** It is well known that irritation in the rectum, as piles, for instance, will produce sympathetic irritation of the bladder or urethra. But this knowledge is often overlooked in practice, and treatment is directed to the branches instead of the root of the disease. A case in point has lately been published in our cotemporary, by Mr. White of Bath, whose attention has long been directed to affections of the rectum in particular.

In September last, a merchant captain, 45 years of age, complained to Mr. W. that he had been afflicted upwards of two years with violent spasms of the urethra, accompanied by considerable difficulty in making water. He had been sounded, but no stone was discoverable in the bladder. One metropolitan surgeon prescribed opium, and considered the disease irritability of the urethra—another passed a bougie, and pronounced the existence of stricture. No abatement of the complaint occurred. During a sea voyage the symptoms became aggravated. On returning to Bristol, a surgeon there examined the rectum, and found a stricture in that canal. Coming to Bath, he consulted Mr. White who, on introducing a bougie, found the rectum extremely irritable and much contracted. There were two strictures—one about four inches from the extremity of the gut—the other seven or eight inches high, which hardly permitted the passage of a seventh-sized bougie. His general health was good ; but his bowels had been obstinately constipated for several years, seldom having an evacuation oftener than once in three or four days. He rarely passed a figured motion, and when he did so, it was very small and flat. After every alvine evacuation the spasms of the urethra came on, though not nearly so severe as during and after making water. During the day he felt very frequent desire to urine, discharging only a very small quantity at the time. Sometimes these spasms were so severe that he was obliged to sit the greater part of the night with a *pot de chambre* between his legs, occasionally passing a few drops of urine, which were sometimes followed by drops of blood. He was directed to take some castor oil every night, and the rectum bougie was regularly persevered in, gradually increasing its size. In about three months the patient was able to pass the largest sized bougie himself, and he left Bath in a very comfortable state, having obtained considerable relief, which he had previously sought in vain.

---

9. *Tracheotomy in diseased Larynx.*† A man, 36 years of age, suffered for several months, in the year 1820, with ulcerated sore throat, supposed to be syphilitic, and which was healed by nitric acid and small doses of mercury. The ulceration occasionally returned, and was removed by the same remedies. Latterly the membrane of the larynx had been frequently affected with inflammation, giving rise to hoarse-

* Mr. White, of Bath. Med. and Phys. Journ. July, 1825.

† Mr. Goodeve, Surgeon to the Clifton Dispensary. London Med. Journal, July, 1825.

ness and some difficulty of breathing. At one period there was acute inflammation about the cricoid cartilage, which was relieved by leeching and blistering. Under pil. hyd. and decoctum sarsæ, he seemed to get well; but he became subject to violent paroxysms of suffocation, in one of which he seemed at the point of death, when a piece of bone was expelled through the glottis with great force. It appeared to have come from the triangular bone of the sternum, where there had been swelling and inflammation some time previously. After this, his voice and respiration improved. Fumigations of cinnabar and a course of the blue pill set his health up once more. But afterwards the paroxysms of dyspnœa returned, and became gradually more aggravated. In fact, the disease of the larynx gained ground, and our author began to foresee that tracheotomy would be the ultimate resource. Early on the morning of September the 25th, Mr. G. was summoned to his patient, who was reported to be dying. On arriving, he found him somewhat recovered from the severest paroxysm he had ever experienced; but still his breathing was laborious and difficult. Venesection had afforded relief. Mr. G. not being provided with a canula, was obliged to put off the operation till 3 in the afternoon, when he found his patient supported in a chair and quite insensible. No pulse could be felt at the wrist, and it was hard to say whether he breathed or not. His face was suffused with blood, and his lips livid. The trachea was instantly laid open by a longitudinal incision, and a circular piece, half an inch in diameter, was cut out by means of a scalpel. The air rushed in, and the patient's breathing quickly improved. After 20 minutes a canula was introduced, and caused much irritation at first, with a convulsive cough, which expelled much bloody mucus. This irritation became less and less, as the repetitions of the introduction took place. And in an hour he could bear the canula very well. This tube he wore more than six months, without inconvenience; he was then able to lay it aside, and his voice was quite restored. This is the second instance of a tube being long worn in the trachea. Mr. Price, of Portsea, has now worn a tube about ten years, and breathes almost entirely through it. He enjoys good health.

We expect to see the day when the operation will be often performed in ulcerations of the larynx, so as to give the said ulcerations time to heal, and the cartilages to exfoliate. We have no doubt that many cases of laryngeal phthisis might be saved by this operation. Ulcerations of the larynx would heal, when the parts were quiet and no air passing, which would otherwise go on from bad to worse, and destroy the patient's life.

P. S.—We understand that Mr. Carmichael has lately performed tracheotomy successfully, in a case of venereal ulceration of the larynx, where the patient was pronounced beyond all hope of life.

10. *Treatment of Ganglion.** Dr. Cumin, surgeon to the Lock Hospital, Glasgow, proposes an operation for ganglion, in some respects novel. The more usual method is to rupture the sac by a blow of a book or other solid body. But this, Dr. C. thinks is a clumsy and unsurgical proceeding. Our author's method is to draw the skin to one side, and then, by pushing in a cataract needle obliquely, to slit the sac of the ganglion freely. The skin is then to be brought to its natural position, forming a kind of valve, and the contents of the ganglion to be squeezed out into the adjacent cellular tissue. Pressure is to be applied; and when the fluid accumulates, it is again to be pressed out, and so on, till the secretion ceases altogether. A case is related in illustration, and our author informs us that he has since treated several others in the same way.

A nearly similar method is described by Jourdan. He directs the skin to be drawn to one side, and the cyst to be divided with the point of a lancet. The contents are to be squeezed out, and the skin to be brought to its natural site, so as to form a valve. Pressure is then to be made, and the operation to be repeated, if necessary.†—The only difference between these two surgeons consists in the form of the instrument and the disposal of the fluid. Dr. C. squeezes it into the surrounding cellular tissue—Jourdan squeezes it out altogether. Now as the wound is made on the same principle in both cases—namely, in a valvular way—we conceive that the fluid, in the first instance, might just as well be squeezed out, if it will come out easily, as into the cellular membrane in the vicinity of the ganglion. The two plans, however, are so nearly alike in principle, that it is not worth while to discuss the superiority of one over the other.

---

11. *Amputation at the Hip.* This terrible operation has been performed, not long since, by Professor Walther, of the University of Bonn, on the Rhine. The subject was a peasant, 21 years of age, who, for six or seven years, had been harrassed with ill-conditioned abscesses in the thigh, by which his constitution was much deteriorated. In this state, a cart wheel passed over the diseased member, and broke the femur in two places, splintering the bone, &c. Having no regular surgical assistance, the whole thigh became a mass of disease.—Hectic fever, night sweats, and colliquative diarrhœa came on—and in this deplorable condition he was carried to the Surgical Hospital of Bonn. Every attention was paid to the unfortunate patient, but no improvement had taken place at the end of six days, except the reduction of pain, and the removal of some splinters. After some hesitation on the part of the surgeon, it was determined to amputate at the hip-joint, as the only means of saving the patient's life.

---

* Dr. Cumin. Ed. Journal, July, 1825.

† See Article Ganglion in the Dictionnaire des Sciences Medicales, tom. xvii. page. 313.

The patient being placed in the usual position, an assistant compressed the artery as it passes over the pubis. Professor Walther then grasped a mass of the integuments, &c. on the outer side of the hip-joint, and thrust a double edged and long pointed knife three inches below the anterior superior spine of the ilium, perpendicularly through the skin, fascia, and tensor vaginæ femoris, down to the neck of the femur. The knife was then directed round the bone, in a direction outwards and backwards, and its point pushed out three inches or better behind the great trochanter, the point and handle of the knife being now nearly parallel, or forming a line at right angles with the thigh bone. The edge of the knife was next directed somewhat upwards, in order to clear the trochanter, and then downwards along the outer side of that process and the upper part of the femur itself, bringing it out about two inches below the base of the great trochanter, thus forming the first flap of the operation, bearing an oval shape, and consisting of integuments, fascia, tensor vaginæ muscle, and portions of the gluteus maximus and medius. No vessels requiring the ligature were divided in this part of the process. Indeed, Professor Walther observes, that if this flap be made in the dead injected limb, the only vessels divided will be branches from the external circumflex artery of the thigh—the femoral artery and vein being full an inch from the anterior edge of the flap, whilst the ischiatic artery and nerve will be found close to, but not involved in, the posterior part of the wound.

The first flap being raised, and turned back by an assistant, then the capsule of the joint became exposed at the angle of the wound. This was easily opened by a bistoury, and the head of the bone was then disarticulated, by carrying the limb horizontally inwards, until it formed a right angle with the body, at the same time turning the member so that the toes and front of the knee were directed upwards. This last manœuvre is considered of much consequence by the operator, as greatly facilitating the disarticulation. The ligamentum teres being rendered tense in the above position, was cut by means of a small knife carried into the joint. A single-edged amputating knife was now passed into the joint, behind the head of the femur, while the assistants gradually changed the position of the limb from a state of adduction to a nearly extended one:—the introduction of the knife and the change of position in the member being simultaneous, and proceeding *pari passu*. The knife was now carried along the inner side of the femur for a space of two inches, avoiding contact with the little trochanter, by slightly raising the edge of the instrument. By this process the great muscles were separated from the bone, the femoral and even the ischiatic vessels as yet remaining intact. An assistant now placing both thumbs in the wound, close to the almost liberated femur, grasped with the remaining four fingers of each hand, the isthmus of soft parts at the inner and upper part of the thigh, thus preventing the flow of blood through the great vessels. The amputation knife being now two inches below the trochanter minor, was directed obliquely downwards and inwards, incising successively the muscles, vessels, nerves, and skin. This part of the operation appears to Professor Walther as the easiest to the operator, though the most painful to the patient. The next step was, obviously enough, to tie the vessels with all expedition. This was easily effected, without more loss of blood than in a common amputation. The femoral artery, the profunda, the femoral vein, the obturator artery, the external circumflex, the ischiatic, and six smaller vessels were tied. The ligatures were cut off close to the knot. The flaps admitted of coaptation without any violence, the surfaces mutually covering each other, and the edges of the flaps coming into nearly uniform contact. Adhesive straps and the usual dressings completed the operation, which the patient bore well. In the afternoon of the same day he had a rigor, which was removed by warm wine and ether. In the night he had some hours of broken sleep, and did not complain in the morning of any particular pain. The dressings were soaked through by a copious watery secretion. The diarrhœa had now ceased since the operation, and the profuse perspiration had diminished. His spirits were good, and the vital functions little disturbed. On the fourth day the dressings were removed, and the inner flap was found somewhat retracted, the wound gaping a little. Incipient union, however, was visible at the angles. Appearances improved during the fifth and sixth days—laudable pus and healthy granulations having appeared.

Unfortunately a gangrenous ulcer which the patient had on the sacrum previous to the operation, now disturbed the progress of the cure. A new accession of fever, with rigors and low delirium set in, and produced great anxiety in the minds of the medical attendants. He died tranquilly on the morning of the eleventh day.

On dissection, all the organs of the chest and abdomen were found perfectly sound. There was no unhealthy appearance on the surface of the wound beyond what might be expected from the changes *post mortem.* In the arteries, the usual plugs of lymph were found behind the ligatures, and the same in the femoral vein, but no inflammation of its inner surface. The German Professor offers some critical remarks on the modes of operating at the hip-joint recommended by Larrey, Guthrie and others, drawing a parallel naturally enough in favour of his own.

This criticism, however, on Mr. Guthrie's method of operating, is, to say the least of it, so unfounded, that it leads us to suspect the Professor never read his work, but has reasoned from report. There is no objection made against Mr. Guthrie's operation that does not more forcibly apply to his own, in corroboration of which assertion, we confidently refer the surgical reader to the operation as performed by Mr. Guthrie on Francois de Gay* after the battle of Waterloo, and who is now living in the *Hotel des Invalides* at Paris.

The operation will be best performed in the following manner:—The patient should be laid on a low table or two field panniers placed to-

* See Guthrie on gun-shot wounds and on amputation of the hip-joint, &c. &c. page 332.

gether, covered with a folded blanket to prevent the edges giving pain, and properly supported in a horizontal position—an assistant, standing on the opposite side and leaning over, should compress the artery against the brim of the pelvis, with a firm hard compress of linen, such as is generally used before the tourniquet; he should also be able to do it with his thumb behind the compress, if it be found insufficient. The surgeon, standing on the inside, with a strong-pointed amputating knife, of a middle size, makes his first incision through the skin, cellular membrane, and fascia, so as to mark out the flaps on each side, commencing about four finger's breadth, and in a direct line below the anterior superior spinous process of the ilium in a well-sized man; and continuing it round in a slanting direction, at an almost equal distance from the tuberosity of the ischium, nearly opposite to the place where the incision commenced. Bringing the knife to the outside of the thigh, he connects the point of the incision where he left off with the place of commencement, by a gently curved line, by which means the outer incision is not in extent more than one third of the size of the internal one. The integuments having retracted, the glutæus maximus is to be cut from its insertion in the linea aspera, and the tendons of the glutæus medius and minimus from the top of the trochanter major. The surgeon now, placing the edge of the knife on the line of the retracted muscles of the first incision, cuts steadily through the whole of the others, blood-vessels, &c. on the inside of the thigh. The artery and vein, or two arteries and a vein, if the profunda is given off high up, are to be taken between the fingers and thumb of the left hand, until the surgeon can draw each vessel out with the tenaculum, and place a ligature upon it. Whilst this is doing, the assistants should press with their fingers on any small vessels that bleed. The surgeon then cuts through the small muscles running to be inserted between the trochanters, and those on the under part of the thigh, not yet divided; and, with a large scalpel, opens into the capsular ligament, the bone being strongly moved outwards, by which its round head puts the ligament on the stretch. Having extensively divided it on the fore part and inside, the ligamentum teres may now be readily cut through—the head of the bone is then easily dislocated, and two or three strokes of the knife separate any attachment the thigh may still have to the pelvis. The vessels are now carefully to be secured. The capsular ligament, and as much of the ligamentous edge of the acetabulum ought to be removed as can readily be taken away. The nerves, if long, are to be cut short—the wound well sponged with cold water, and the integuments brought together in a line from the spinous process of the ilium to the tuberosity of the ischium. Three sutures will, in general, be required, in addition to the straps of adhesive plaster, to keep the parts together; the ligatures are to be brought out in a direct line between the sutures, a little lint and some compresses are to be placed over the wound and on the under flap, to keep it in contact with the cotyloid cavity, and assist the union of the parts—a piece of fine linen is to be laid over them, and the whole retained by a calico bandage, put round the waist and brought over the wound.

It is recommended to pare the cartilage from the bone; and if this could be readily done, we would agree to it, but the cartilaginous surface, of the acetabulum is not to be cut away without much difficulty and some time, which cannot be spared; for we consider the success of the operation to depend very much upon the quickness with which it is performed, not on account of hæmorrhage, but to avoid the shock the constitution receives from the continued exposure and irritation of so large a surface in the immediate vicinity of the trunk of the body. It is proved, by experience, to be unnecessary at the shoulder-joint, and will, we think, be found equally so at the hip.

Union by the first intention is to be wished for in a great degree, as lessening the surface of the wound; but as all the parts beneath the skin cannot unite, and especially about the acetabulum and the inside of the glutæus muscle, it is not advisable to let the skin adhere on the middle and lower part of the stump; for, as the parts deep-seated must suppurate and granulate, a fair opening for the discharge should be preserved, and collections of matter in any part should be carefully guarded against by gentle pressure, compress and bandage.

In Mr. Guthrie's successful case, the particulars of which have been already referred to, the situation of the posterior shot-hole prevented, in a great measure, the inconvenience likely to arise from any retention of matter; it became necessary, however, to make an opening at this part during the cure, and, from a due consideration of all these circumstances, he now recommends that an opening should be made through the outer flap at the period of operating, immediately below the acetabulum, into which a piece of lint should be introduced until suppuration is duly established, when it may be readily kept open until all fear of the collection of matter has subsided or been removed. If it should collect although every precaution be taken to prevent it, an opening should be made at a dependent part with as little delay as possible.

By comparing these two methods, it will be perceived that Professor Walther's outer flap is a direct imitation of Mr. Guthrie's, with the disadvantage of being larger, deeper, and more liable to give rise to hæmorrhage. Mr. Guthrie's external flap being a division *solely* of the integuments, and the insertion of the glutei muscles, for the purpose of connecting the extremities of the first incision and of facilitating the disarticulation of the bone. In regard to the vessels, we do not consider it necessary to place ligatures on any of them, except the femoral artery and the profunda, until the limb is separated from the body, and on these only, if the compression should be imperfect, and we do not think Professor Walther's reasoning in favour of disarticulating the bone from the outside before making the inner flap in any way satisfactory. On the contrary, it appears to us to render the operation much more complicated and not less dangerous. We also differ with Professor Walther as to the propriety or necessity of performing the operation in this case; for as dissection proved the head, the cervix, and the trochanter major and minor of the bone to be sound, a simple flap or common amputation sawing through the great trochanter would have been sufficient.

12. *Herpes Genitalis.* Herpes genitalis is a common affection. Simple in itself, of an herpetic nature, and, therefore, pursuing a determinate course, it is easily understood; but, if misconceived or mismanaged, or thought to be syphilitic, and treated as such, the consequences may embitter existence. This is the first attempt to name and class the affection. Royston and Bateman have written on præputial herpes, without intimating that it takes a wider range.

Herpes genitalis is assumed as a generic term, and implies a slight and circumscribed inflammatory affection of the male genitals, proceeding to ulceration, advancing regularly towards a cure, and without leaving a cicatrix. There are three species, viz.—1. *Herpes præputii;* 2. *Herpes glandis;* 3. *Herpes scroti.*

1. *Herpes Præputii.* This species presents two varieties—1*st. Herpes præputii interni*—2*nd. Herpes præputii externi.*

1. *Herpes Præputii Interni.* Heat and itching are experienced under the præpuce, and, upon examination, a reddish and elevated spot of the size of a silver penny, or larger, is visible. In a few hours, according to the severity of the attack, which is various indeed, three, four, or more, watery points are apparent. Next day, these points coalesce, and constitute an ulcer, which is rather painful, and exhibits a whitish surface. Under judicious management, the heat and other unpleasant sensations cease, the ulcer becomes clean and red, and soon heals. The time of cure vacillates between three, seven, twelve, or twenty days, or even longer; for, in severe cases, the præpuce is considerably tumefied, and the heat and pain distressing. *Ceratum calaminæ*, spread on lint, imparts immediate ease, and always succeeds. No medicine is needed, and he that employs dry lint, or any other application, will repent his rashness.

2. *Herpes Præputii Externi.* The stages of this are less obvious, because external, and irritated by the shirt. By mismanagement, it often degenerates into a troublesome ulcer, which may be, and has been, mistaken for a chancre. No treatment is required. If irritation be guarded against, the discharge forms an incrustation, and, if left to itself, this incrustation will slowly separate, and leave the subjacent part healed. If this salutary incrustation be prevented, great mischief ensues, and the ulcer extends, creating doubt and anxiety.

3. *Herpes Glandis.* Here the affection is more irritable, and the ulcer proportionably obstinate. The cure is longer, yet *ceratum calaminæ* on lint is the only proper application, since every other is injurious.

3. *Herpes Scroti.* Herpes scroti begins at the lower part, and near the rapha. There are heat and itching, and slight induration is felt. If any application be made, it becomes a tedious and annoying ulcer. No means are to be adopted: an incrustation will arise, which will gradually crumble, and the part underneath will be sound.

For every species or variety of herpes genitalis, medicine is unnecessary; and the exciting cause of it is irritability of the urethra. The foregoing observations are the result of an experience of fifteen years, on several hundred cases.

——— "quæque ipse miserrima vidi,
"Et quorum pars magna fui." AMICUS.

---

13. *Idiotism of many Years Continuance, cured by the Extirpation of the Clitoris.** The following case was communicated to the Journal just named, by a physician now practising at Berlin, and, although he has not allowed his name to appear, Professor GRAEFE states, in a note, that he is perfectly satisfied with the authority on which the case is presented to the public, and pledges himself as to its correctness. The family of the patient is very respectable, and now resides at Potsdam, near Berlin. The early history of the case was obtained principally from the young lady's mother, and is, in substance, as follows:—

"A. D. was born in 1807, and was, at the time of birth, a very healthy and well-formed child. It was soon committed to the care of a very healthy nurse, with whom it thrived well, was active and cheerful. Nothing particular occurred until the child was about fourteen months old, when it was seized with a violent diarrhœa, in consequence of being allowed to remain with the nurse, who had an attack of fever, and owing to the improper treatment of the practitioner, this complaint continued, more or less alternating with constipation, for eight months. The child was exceedingly weakened, and remained so for a long time, so that it was not until the fourth year that it was able to walk. The development of intellect, at this period, was observed by the mother to be somewhat restricted, and, soon after this time, it was supposed, by the latter, that the disease began. At this period, also, the appetite was very voracious, whilst the greatest aversion was shown to fluids of all kinds. In the year 1812, Dr. Bremer was consulted, who imagined that the disturbed state of the mental and physical powers was owing to the irritation of worms in the alimentary canal, anthelmintics were prescribed, and some worms discharged, but the state of the little patient remained just as before. Professors Wolfars and Horn were consulted in 1816, and Dr. Becker of Leipzig in 1818, who, after having tried electricity, alterative medicines, lotions, and injections of various kinds, gave up the case as incurable."

This little unfortunate had now reached its tenth year, and the marks of an impaired state of intellect, which had begun to shew themselves at four, had in no measure diminished, but, on the contrary, increased. The child could not recognize its parents, brothers, or sisters, and if any toys were given to it, they would be immediately destroyed. She

---

* Graefe's and Walther's Journal der Chirurgie. Subenter Band, Erstes Neft.

would sit for a considerable time in a corner of the room, with her elbows resting on her knees, and her fingers in her ears, with her eyes closed, and the saliva flowing from her half-open mouth: at other times, she was very restless, and, occasionally, very noisy and obstreperous, seizing any thing she could lay her hands on, and throwing it out of the window; her taste was, also, very imperfect, and she would eat what to others was very unpalatable food, with the greatest relish. Her speech was so impaired that she could not make herself intelligible, the stools and urine were passed involuntarily, and the things which ordinarily engage the attention of children remained by her unnoticed. Music appeared to interest her a little, and she could distinguish the different sounds; the sleep was as much as is usual, but never continued long at a time. During the years 1820 and 1821, the patient was placed under the care of Dr. N———, who took a new view of the case, and considered that there must be some cause which had not yet been discovered. By an attentive examination and observation, he ascertained that the patient was addicted to a disgusting and solitary vice, and this increased afterwards to such a height that she scarcely abstained from it in the presence of the other sex. The opinions which influenced Dr. N. in the treatment of the case were principally these—he considered that the practice alluded to very much tended to maintain the state of imbecility of the patient: that the excitement of the mental energy by powerful contra-stimulation, would be likely to restrain such a practice, and that the diet should be strictly regulated. Mechanical restraints were used, in order that she might not be able to indulge in such a practice, and, during the day time, an apparatus was contrived, which caused great pain whenever she attempted to skulk away in the corner, as before mentioned: camphor was also given, in small and large doses, for a considerable time, which somewhat diminished her vicious propensity; but, notwithstanding every possible attention, she sought every opportunity of eluding the watchfulness of her keepers. Finding this still to be the case, it was agreed, after consultation with Professor Rust, that powerful counter-irritation should be employed, and, after the head had been shaved, the actual cautery was applied just over the tuberosity of the occipital bones; the wound was made about the size of a half-crown. The patient did not complain of pain during the operation, and ate, with great greediness, a biscuit that was given her. The wound was kept open for more than two months, by peas and irritating dressings, and during the early part of July, warm baths and cold affusions were repeatedly and alternately used. Under this mode of treatment, a considerable improvement took place, and the disposition to her former habits sensibly diminished. She began to give some signs of returning intellect, and it was remarked, that when punished she shed tears, which she had never before done; she began, also, to distinguish objects, and to understand their several qualities. Thus she continued for some time, but her former propensities returned, and those as violent as before. Warm baths, nauseating and emetic medicines, with other measures, to lessen the force of the circulating system were employed, without producing any decided effect. The most prominent symptoms continued to be the "desiderium impurum" before-mentioned, and thus the case remained up to the commencement of June, 1822.

After having consulted all the authorities on this subject, it was found that the French had represented the extirpation of the clitoris to be the most effectual remedy, and one totally devoid of danger. The operation was performed on the 20th of June, by Professor Gräefe, with great ease and celerity, by means of a scissors; the hæmorrhage was very slight, and was readily suppressed by washing the part with cold water. The result of the operation was regarded with the greatest anxiety, and it was, therefore, with great pleasure that it appeared to be so favourable. The patient gave proofs of an increase of mental power; in the month of July, she learned to write, or rather, commenced to learn writing, and began to express herself correctly; but when she could not make herself thoroughly understood, she endeavoured to illustrate her meaning by a graphic representation. In September following, some unpleasant symptoms again made their appearance, for which a blister was applied to the back; this was kept open for some time, and she soon got well. In October, a slight disposition to her former propensity manifested itself, on which account the place on which the blister had been before applied was annointed with tartar emetic ointment, which soon caused it to disappear. From the time named to the present, the patient has been gradually improving, mentally and corporeally, and, up to the time when this account was closed, had not manifested the slightest inclination to her former vices. She has learned to read and write, and pays the greatest attention to what her parents and others may say, and she is, to use the words of the narrator of the case, altogether "a new creature."

---

14. The Copenhagen Needle Patient.* In 1822, Professor Hecholdt published an account of this very extraordinary case, in a pamphlet entitled '*Observatio de Affectibus Morbosis Virginis Havniensis, cui plurimæ acus e Variis Corporis partibus excisæ et extractæ sunt.*' The number of needles extracted up to the time of the appearance of Dr. H's book was 295, since which period, one hundred and five more have been removed. It was for the purpose of communicating this additional information, and likewise for the purpose of making the case more generally known, that Dr. Otto sent the present version of the case to the Journal above named. Dr. Otto observes, that he hopes that the circumstantial manner in which the case is given will remove every doubt from the most sceptical, but, that if any further corroboration be necessary, he can appeal to thirty medical practitioners at Copenhagen, who have seen the patient at different periods of the complaint, and she may

---

* *History of a Patient from whom four hundred needles were extracted* From Hecker's Litterarische Annalen der Gesammten Heilkunde, July, 1825.

at the present moment be seen by every one, at Frederick's Hospital, in that place.

Rachel Hertz had lived in the enjoyment of good health up to her fourteenth year; she was then of a fair complexion, and rather of the sanguineous temperament. In August 1807, she was seized with a violent attack of cholic, which induced her to apply to Professor Hecholdt, and this was the first acquaintance which the Professor had with the case. From that time to March 1808 she experienced frequent attacks of erysipelas and fever, which left her in a very debilitated state. Many symptoms of an hysterical character showed themselves, but which the ordinary remedies failed to remove. From March to May 1809, a period of fourteen months, she suffered in this way from repeated and violent hysteric attacks, accompanied with, or rather followed by, fainting, which sometimes continued so long, that people considered her dead. Occasionally she was attacked with epileptic fits, at other times with drowsiness and hiccough, and sometimes with delirium. During the paroxysms of her madness, she delivered, with a loud voice and correct enunciation, long passages from the works of Göthe, Schiller, Shakspeare, and Othlenschläger, just as accurately as any sane person could do, and although she kept her eyes closed, she accompanied the declamation with suitable gesticulations. The delirium continued to increase until it assumed a very alarming height; she gnashed her teeth, kicked about, and fought with whatever came in her reach, and disturbed with her ravings, not only her own household, but the whole neighbourhood. The patient was next the subject of a violent attack of hæmatemesis, which returned, in a greater or less degree, for several months, notwithstanding the employment of the usual remedies. In January 1809, she complained of severe pain in the lower part of the abdomen, about the sigmoid flexure of the colon, attended with obstinate constipation and severe ischuria. It was discovered, by an examination of the rectum, that a contraction existed there, and the accumulation of the feculent matter above the constricted part was supposed to explain the production of the ischury. The bladder could not be emptied without the use of the catheter or bougie, the introduction of which was exceedingly difficult. Purgatives of various kinds, injections without number, the aromatic hip bath, fomentations of the abdomen, and repeated cold affusions were used without success, as regarded the ischury, although the constipation, and the major part of the hysterical symptoms disappeared. For two years, the daily introduction of the catheter was necessary. In March 1809, the state of the patient was somewhat improved; she was able to rest better at night, and her behaviour during the day was more tranquil. This did not long continue: the tranquillity soon degenerated into stupor, which became daily more profound. At the middle of the day, she remained as if dead, without sense or motion, with the respiration so slow and feeble that it could with difficulty be detected. For about an hour after noon, she plunged from this state of stupor suddenly into a raving madness, attended with dreadfully severe convulsions; a similar paroxysm made its appearance at night. She took nothing to eat or drink for a whole week, in which time the bowels were only relieved once, and then without her knowledge.

In May, 1809, Professor Collisen was consulted, who, during the lethargic state of the patient, recommended snuff to be pushed up her nostrils, the effect of which was so favourable that, without sneezing, she soon came to her senses. She complained of nothing during that day, and the snuff frequently produced equally good effects, for a time only. The delirium continued from May 1809, to December 1810, with little variation of importance, and then gradually subsided. At the close of November, 1810, the patient sunk into a state of great debility, and remained for eight days as if dead, the extremities were cold, the countenance cadaverous and the respiration slow and very feeble; when she emerged from this state, a slight fever succeeded, and it was observed that she was incapable of moving her right side. Here is a seeming inconsistency in the narration of the case; for we are told that, for the next two years, she enjoyed good health. In April, 1813, she had the measles, which disappeared in the usual time. In July, an intermittent fever made its appearance, and, in August, the old complaint of hæmatemesis returned. All these misfortunes were removed, and this walking hospital was once more permitted to be purified; for it is stated that, from the end of November to June, 1813, she remained well. Nothing particular occured from that time to May 1816, except the formation of a carbuncle in the thigh, but she was then seized with violent pains in the left hypochondriac region, with vomiting of blood, from which she again recovered, and remained free from any particular complaint until January 1819, when severe cholic pains made their appearance, with fever, vomiting of blood, and purging of black fæcal matter, from which it was considered impossible that she could recover—but recover she did. On examination of the abdomen, a large tumour was found, having three distinct elevations just below the umbilicus. To this tumour, emollient cataplasms were applied, without producing much benefit, and the worn out and desperate condition of the patient induced Professor Hecholdt to lay open the tumour by a deep incision. It was expected that a copious discharge of pus would follow, but no pus came, aud the bleeding was very slight. When the wound was examined with a probe, a curious sensation was communicated to the hand, just as if a metallic body had been thrust against the probe; this was repeated, a forceps was introduced, the substance was laid hold of, and, to his great astonishment, out came *a needle.* The extraction of this needle produced some alleviation of the sufferings of the patient, but it was of very short duration; great pain with vomiting of blood returned, another tumour appeared in the left lumbar region, the touching of which caused great uneasiness. On the 15th of February, an incision was made into it, and another black oxydized needle drawn out. It would be tedious and uninteresting to describe the situation and appearance of every subsequent tumour; suffice it to say that, from the 12th of February 1819, to the 10th of August

1820, a period of eighteen months, severe pains, followed by tumours, were felt in various parts of the body, from which *two hundred and ninety-five needles* were extracted, viz.—

From the left breast, 22—from the right breast, 14—from the epigastric region, 41—from the left hypochondriac region, 19—from the right hypochondriac region, 20—from the umbilicus, 31—from the left lumbar region, 39—from the right lumbar region, 17—from the hypogastric region, 14—from the right iliac region, 23—from the left iliac region, 27—from the left thigh, 3—from the right shoulder, 23—between the shoulders, 1—from under the left shoulder, 1—total 295.

Some of them were broken, some were without points, some were small, others large. They made their appearance at considerable intervals; sometimes one showed itself every day, then for a week no more could be detected, and, occasionally, a month intervened from the extraction of one to the extraction of another. The patient, during the greater part of this time, was so weak as to be obliged to keep her bed, and she said that she did not feel any pain until the needles approached the surface of the body. From August, 1820, to the 8th of March, 1821, no more needles had made their appearance, and it was not not until after the appearauce of Professor Hecholdt's account of the case had been published in 1822, that any more were extracted. This part of the history is continued by Dr. Otto, who goes on to say, that a large tumour formed in the right axilla, from which, between the 26th of May and the 10th of July, 1822, no less than *one hundred needles* were taken out! The patient for some time after was the subject of a diabetes, which again threatened her life, but from which, as from her former desperate attacks, she ultimately recovered. From the 1st of July, 1822, to the 10th of December, 1823, five needles were at different times extracted, making the total number of FOUR HUNDRED!! The patient has amused herself during her convalescence by learning Latin, and writing a journal of her own case. She is at present living at Frederick's hospital, at Copenhagen, and enjoys good health.

---

15. *Acupuncturation.** It is by no means universally believed that there are any good effects resulting from acupuncture, beyond those produced on the patient's imagination. In medicine we are very prone to doubt every thing for which we cannot satisfactorily account. But this is a dangerous scepticism in many cases; for, in truth, we can account for very few of the phenomena that are daily presented to our eyes. If, one hundred years ago, a man had proclaimed that an ear of rye would make a woman miscarry, he would have been laughed at as a visionary or a knave. Yet the thing is proved beyond the possibility of doubt. At present it is the fashion among the knowing ones of this Metropolis to rank acupuncture and moxibustion with metallic tractation and animal magnetism. We have not seen enough of either process to authorize a dictum on the occasion—and therefore we shall con-

* Dr. Webster. London Med. Journ. July, 1825.

tent ourselves, in the present stage of the enquiry, with making reports, from time to time, of such histories as appear well authenticated.

Dr. Webster mentions the case of a female, who had been bled in the arm for pain in the head. At the moment the vein was opened, she felt excruciating pain, and, whilst the blood was flowing, she fainted. The wound healed kindly, and during the week she was free from pain. At the end of that time she suddenly experienced a severe pain, proceeding from the bend of the elbow, and extending down the forearm, in the course of the inner cutaneous nerve, to the hand. This pain was most severe about two inches below the inner condyle of the humerus, and in the muscles composing the fleshy part of the thumb. These symptoms continued and gradually increased for four months and upwards. At the time she was first seen by Dr. Webster, the pain was most excruciating, especially in the upper part of the forearm, and in the cicatrix. Many remedies and applications were tried, but none gave any relief, except a blister that extended from the bend of the arm to the wrist. Acupuncture was therefore determined on. A needle was introduced at two different points in the upper and inner part of the forearm, to the depth of three quarters of an inch. The same was done in the ball of the thumb, until the needle almost penetrated through the hand. The instrument was turned gently round at each introduction, but did not remain longer than 15 seconds at a time. The patient felt little pain in consequence, except when the needle was turning. The original pain continued, without diminution, for two days afterwards. On the third day it was much alleviated. On the fourth she was free from complaint, and remained so.

